# Diagnostic and Clinical Patterns of Newly Diagnosed ANCA-Associated Vasculitis Before and After the 6 February 2023 Kahramanmaraş Earthquakes: A Multicenter Retrospective Study from Affected Provinces

**DOI:** 10.3390/jcm15176649

**Published:** 2026-08-28

**Authors:** Sezgin Zontul, Gezmiş Kimyon, Fuat Albayram, Anıl Göçer, Rabia Sağır, Aylin Dolu Karaca, Elif İnanç, Zeynep Kaya, Mesude Seda Aydoğdu, İpek Balıkçı Çiçek, Gözde Yıldırım Çetin, Servet Yolbaş

**Affiliations:** 1 Division of Rheumatology, Department of Physical Medicine and Rehabilitation, Faculty of Medicine, Inonu University, Malatya 44280, Turkey; 2 Division of Rheumatology, Department of Internal Medicine, Faculty of Medicine, Hatay Mustafa Kemal University, Hatay 31060, Turkey; gkimyon@gmail.com; 3 Division of Rheumatology, Department of Internal Medicine, Faculty of Medicine, Inonu University, Malatya 44280, Turkey; albayramfuat@hotmail.com (F.A.); rabia.sagir@inonu.edu.tr (R.S.); servet.yolbas@inonu.edu.tr (S.Y.); 4 Division of Rheumatology, Department of Internal Medicine, Faculty of Medicine, Kahramanmaraş Sütçü İmam University, Kahramanmaraş 46050, Turkey; anilgocer1990@gmail.com (A.G.); gozdeyildirimcetin@gmail.com (G.Y.Ç.); 5 Division of Rheumatology, Department of Internal Medicine, Adıyaman Training and Research Hospital, Adıyaman 02040, Turkey; nilyaulod@hotmail.com; 6 Division of Rheumatology, Department of Internal Medicine, Van Training and Research Hospital, University of Health Sciences, Van 65300, Turkey; elif.temelli@hotmail.com; 7 Division of Rheumatology, Department of Internal Medicine, Istanbul Training and Research Hospital, Istanbul 34098, Turkey; zeynepkaya00@gmail.com; 8 Division of Rheumatology, Department of Internal Medicine, Malatya Turgut Özal University, Malatya 44330, Turkey; kinaci_seda@hotmail.com; 9 Department of Biostatistics and Medical Informatics, Faculty of Medicine, Inonu University, Malatya 44280, Turkey; ipek.balikci@inonu.edu.tr

**Keywords:** ANCA-associated vasculitis, earthquake, natural disaster, disease activity, multicenter study

## Abstract

**Background/Objectives:** The relationship between large-scale natural disasters and antineutrophil cytoplasmic antibody (ANCA)-associated vasculitis (AAV) remains uncertain. This study compared crude case numbers, diagnostic patterns, and clinical characteristics of newly diagnosed AAV before and after the 6 February 2023 Kahramanmaraş earthquakes in Türkiye. **Methods:** This multicenter retrospective study included patients newly diagnosed with AAV between 6 February 2020 and 5 February 2026 at five tertiary referral centers in four provinces heavily affected by the earthquakes. Equal 36-month pre- and post-earthquake periods were compared. Demographics, AAV subtype, ANCA serology, biopsy-confirmed diagnosis, organ involvement, Birmingham Vasculitis Activity Score (BVAS), laboratory findings, treatments, and recorded outcomes were evaluated. **Results:** A total of 127 patients were included: 58 (45.7%) before and 69 (54.3%) after the earthquakes. The crude number of cases was numerically higher after the earthquakes, but the difference was not statistically significant (*p* = 0.375). AAV subtype, ANCA serology, organ involvement, BVAS, inflammatory markers, renal function, and most laboratory findings were comparable. Biopsy-confirmed diagnoses were significantly less frequent in the post-earthquake period than in the pre-earthquake period (46.4% vs. 70.7%, *p* = 0.010). Treatment patterns were comparable between the two periods. Although the crude proportions of recorded outcomes did not differ significantly, these comparisons were limited by the substantially shorter follow-up duration in the post-earthquake group and should not be interpreted as evidence of comparable prognosis. **Conclusions:** In major tertiary referral centers serving earthquake-affected provinces, crude AAV case numbers and baseline clinical phenotypes were largely comparable before and after the earthquakes. These crude case numbers should not be interpreted as incidence estimates, because reliable population denominators were unavailable after the earthquakes. The significant reduction in biopsy-confirmed diagnoses may reflect post-disaster changes in diagnostic healthcare pathways. These real-world data provide an important regional overview of newly diagnosed AAV after a major natural disaster.

## 1. Introduction

Antineutrophil cytoplasmic antibody (ANCA)-associated vasculitis (AAV) comprises a group of rare but potentially life-threatening systemic autoimmune diseases characterized by necrotizing inflammation, predominantly affecting small vessels. This disease spectrum primarily comprises granulomatosis with polyangiitis (GPA), microscopic polyangiitis (MPA), and eosinophilic granulomatosis with polyangiitis (EGPA), and it may involve multiple organ systems, particularly the kidneys and respiratory tract [1]. Despite substantial advances in diagnosis and treatment, AAV remains associated with considerable morbidity and mortality [2]. AAV is considered a rare disease, with reported incidence varying across geographic regions [3].

Although the etiopathogenesis of AAV has not been fully elucidated, the disease is believed to arise from a complex interplay between genetic susceptibility and environmental exposures [3,4,5]. The emerging evidence suggests that several environmental factors, including silica exposure, air pollution, infections, and various inhalational agents, may contribute to the development of AAV [5,6], promoting neutrophil activation and facilitating the generation of autoimmune responses against antigens such as proteinase 3 (PR3) and myeloperoxidase (MPO), thereby contributing to the initiation of vascular inflammation [4].

On 6 February 2023, two major earthquakes with moment magnitudes of 7.8 and 7.6 occurred approximately nine hours apart in the Kahramanmaraş region of southeastern Türkiye. These earthquakes were considered the most devastating natural disaster in modern Turkish history and among the deadliest earthquakes of the twenty-first century. The disaster directly affected 11 provinces, resulting in more than 50,000 deaths, the destruction or severe damage of hundreds of thousands of buildings, and the displacement of millions of people [7]. In the aftermath of these earthquakes, several factors with potential immunological consequences emerged, including intense exposure to dust and particulate matter from debris, large-scale population displacement, a deterioration in living conditions, an increased risk of infectious diseases, and substantial psychosocial stress [7,8]. These factors may influence immune regulation and potentially contribute to the development or exacerbation of autoimmune diseases. To date, only a limited number of studies have investigated the relationship between natural disasters and AAV, yielding conflicting results. Following the 1995 Great Hanshin (Kobe) Earthquake in Japan, an increase in MPO-ANCA-associated vasculitis was reported [9]. Similarly, the 2011 Great East Japan Earthquake was associated with an increased incidence of microscopic polyangiitis, higher disease activity, and poorer survival outcomes [10]. In contrast, a study conducted after the 2011 Christchurch Earthquake in New Zealand found no significant change in the incidence of AAV [11]. Therefore, the relationship between large-scale natural disasters and the development and clinical characteristics of AAV remains incompletely understood.

Given the rarity of AAV and the major disruption caused by the earthquakes in the affected provinces, real-world data from regional tertiary referral centers may provide useful information on post-disaster diagnostic and clinical patterns. To the best of our knowledge, this is the first multicenter study to evaluate newly diagnosed AAV cases before and after the February 2023 Kahramanmaraş earthquakes. Therefore, we aimed to compare the crude case numbers, diagnostic features, and baseline clinical characteristics of newly diagnosed AAV cases during equal pre- and post-earthquake periods in tertiary referral centers serving heavily affected provinces.

## 2. Materials and Methods

### 2.1. Study Design and Population

This multicenter retrospective observational study was conducted at five tertiary referral centers located in the provinces that experienced the highest burden of mortality, structural damage, and population displacement following the 6 February 2023 Kahramanmaraş earthquakes in Türkiye: İnönü University Faculty of Medicine and Malatya Training and Research Hospital (Malatya), Adıyaman Training and Research Hospital (Adıyaman), Hatay Mustafa Kemal University Faculty of Medicine (Hatay), and Kahramanmaraş Sütçü İmam University Faculty of Medicine (Kahramanmaraş).

The study aimed to compare the crude case numbers, diagnostic patterns, and clinical characteristics of newly diagnosed AAV cases before and after the earthquakes. Medical records of patients newly diagnosed with AAV between 6 February 2020 and 5 February 2026 were retrospectively reviewed. The study period was divided into a pre-earthquake period (6 February 2020–5 February 2023) and a post-earthquake period (6 February 2023–5 February 2026).

Patients aged ≥ 18 years who were newly diagnosed with AAV and classified as GPA, MPA, or EGPA, according to the 2022 American College of Rheumatology/European Alliance of Associations for Rheumatology (ACR/EULAR) classification criteria, were included [12,13,14]. Patients who did not meet the classification criteria for a specific AAV subtype were categorized as having unclassifiable AAV. Patients with a previous diagnosis of AAV and those with insufficient data for diagnostic confirmation or clinical evaluation were excluded from the study.

The study protocol was approved by the Inonu University Non-Interventional Clinical Research Ethics Committee (Approval No: 2026/9604; 10 March 2026). Given the retrospective design of the study, the requirement for informed consent was waived. The study was conducted in accordance with the ethical principles of the Declaration of Helsinki and its subsequent amendments.

### 2.2. Data Collection and Clinical Assessment

Demographic, clinical, and laboratory data were obtained through a retrospective review of medical records. Demographic variables included age and sex. Disease-related variables comprised the date of diagnosis, AAV subtype (GPA, MPA, or EGPA), ANCA specificity [proteinase 3 (PR3)-ANCA or myeloperoxidase (MPO)-ANCA], and organ involvement. Organ involvement was categorized as renal, pulmonary, ear–nose–throat (ENT), neurological, cutaneous, ocular, gastrointestinal, cardiac, musculoskeletal, and other systemic manifestations.

Laboratory parameters included the erythrocyte sedimentation rate (ESR), C-reactive protein (CRP), serum creatinine level, and complete blood count findings. The disease activity at diagnosis was assessed using the Birmingham Vasculitis Activity Score (BVAS) [15]. The total score ranges from 0 to 63, with higher scores indicating a higher burden of active vasculitic manifestations. Treatment-related data, including induction and maintenance therapies, were also recorded. Clinical outcomes, including hospitalization, intensive care unit admission, remission, relapse, dialysis requirement, and mortality, were evaluated.

### 2.3. Statistical Analysis

Categorical variables are expressed as numbers and percentages, whereas continuous variables are presented as the mean ± standard deviation (SD) or median (minimum–maximum), as appropriate. The normality of continuous variables was assessed using the Kolmogorov–Smirnov test. Comparisons between the pre- and post-earthquake groups were performed using the independent-samples *t* test for normally distributed variables and the Mann–Whitney U test for non-normally distributed variables. Categorical variables were compared using Pearson’s chi-square test, Yates’ continuity-corrected chi-square test, or Fisher’s exact test, as appropriate. A single-sample binomial test was used to compare the distribution of newly diagnosed AAV cases between the pre- and post-earthquake periods. Effect sizes were calculated, when appropriate, using Cohen’s d, the rank-biserial correlation coefficient, and Cramér’s V. Associations between continuous variables were assessed using Spearman’s rank correlation analysis. A two-sided *p* value < 0.05 was considered statistically significant for all analyses. Statistical analyses were performed using IBM SPSS Statistics version 26 (IBM Corp., Armonk, NY, USA) and Python (version 3.13.5).

## 3. Results

**Figure 1:**
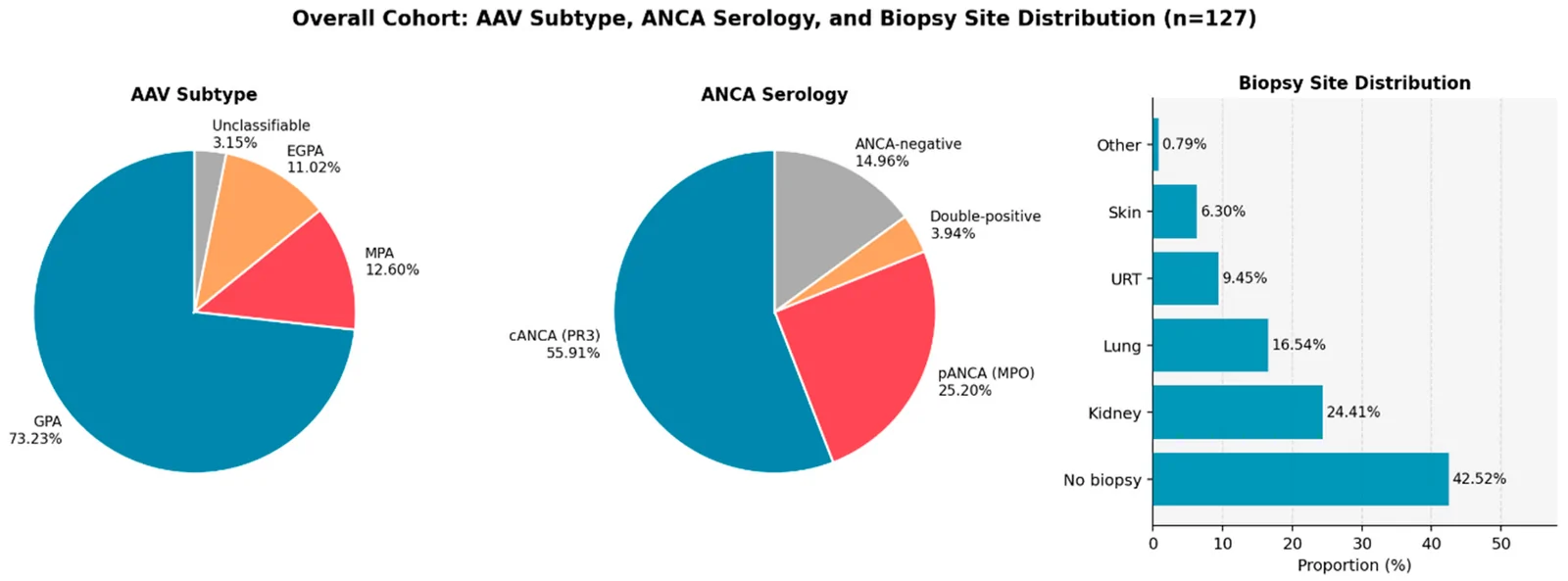
Distribution of ANCA-associated vasculitis (AAV) subtypes, ANCA serological patterns, and biopsy sites in the overall study population (*n* = 127). ANCA, antineutrophil cytoplasmic antibody; GPA, granulomatosis with polyangiitis; MPA, microscopic polyangiitis; EGPA, eosinophilic granulomatosis with polyangiitis; PR3, proteinase 3; MPO, myeloperoxidase; URT, upper respiratory tract.

A total of 127 patients with newly diagnosed ANCA-associated vasculitis (AAV) from five tertiary referral centers located in Malatya, Kahramanmaraş, Hatay, and Adıyaman were included in the study. Of these, 58 (45.7%) were diagnosed during the pre-earthquake period and 69 (54.3%) during the post-earthquake period. Although the number of newly diagnosed AAV cases was numerically higher after the earthquakes, the difference was not statistically significant when the two observation periods of equal duration (36 months each) were compared (*p* = 0.375). The distributions of AAV subtypes, ANCA serological patterns, and biopsy-confirmed diagnoses are presented in Figure 1.

The demographic and clinical characteristics of the study population are summarized in Table 1. No significant differences were observed between the pre- and post-earthquake groups regarding age, age at diagnosis, sex, body mass index, smoking status, AAV subtype, ANCA serology, or center distribution. The follow-up duration was significantly longer in the pre-earthquake group (*p* < 0.001). In addition, the proportion of biopsy-confirmed diagnoses was significantly higher in the pre-earthquake group than in the post-earthquake group (70.7% vs. 46.4%, *p* = 0.010).

The patterns of organ involvement in the pre- and post-earthquake groups are presented in Table 2. No statistically significant differences were observed between the groups regarding pulmonary, upper respiratory tract, renal, neurological, cutaneous, musculoskeletal, ocular, gastrointestinal, cardiac, or other organ involvement (all *p* > 0.05). Although upper respiratory tract involvement was more frequent in the pre-earthquake group than in the post-earthquake group (67.2% vs. 49.3%), the difference did not reach statistical significance (*p* = 0.063).

The laboratory parameters at diagnosis and BVASs are presented in Table 3. The BVASs ranged from 1 to 40 in the overall cohort. No significant differences were observed between the pre- and post-earthquake groups regarding the BVAS, ESR, CRP, serum creatinine, white blood cell (WBC) count, hemoglobin, albumin, alanine aminotransferase (ALT), or aspartate aminotransferase (AST) levels (all *p* > 0.05). However, the platelet counts were significantly higher in the pre-earthquake group than in the post-earthquake group (347.5 vs. 287.0 × 10^3^/µL, *p* = 0.029).

The treatment approaches and clinical outcomes are summarized in Table 4. The distribution of agents used for induction and maintenance therapy was comparable between the pre- and post-earthquake groups. Likewise, no statistically significant differences were observed in the crude proportions of recorded outcomes, including remission, relapse, dialysis requirement, and mortality, between the two periods (all *p* > 0.05).

The correlations between the BVASs and laboratory parameters are presented in Table 5. In the overall cohort, the BVAS demonstrated moderate positive correlations with CRP (r = 0.423, *p* < 0.001) and serum creatinine (r = 0.426, *p* < 0.001). In addition, weak positive correlations were observed between the BVAS and ESR (r = 0.295, *p* = 0.001), as well as the WBC count (r = 0.358, *p* < 0.001). Conversely, the BVAS showed weak negative correlations with the hemoglobin (r = −0.371, *p* < 0.001) and albumin levels (r = −0.374, *p* < 0.001). No significant correlations were identified between the BVAS and ALT or AST levels. Similar correlation patterns were observed in the pre- and post-earthquake subgroups.

## 4. Discussion

In this multicenter retrospective study, we evaluated the crude case numbers, diagnostic patterns, and clinical characteristics of newly diagnosed ANCA-associated vasculitis (AAV) before and after the 6 February 2023 Kahramanmaraş earthquakes in four heavily affected provinces. The main findings were as follows: (i) the crude number of newly diagnosed AAV cases was numerically higher in the post-earthquake period, but this difference was not statistically significant; (ii) the AAV subtype distribution, ANCA serology, organ involvement, BVAS, and most laboratory parameters were largely comparable between the pre- and post-earthquake periods; (iii) biopsy-confirmed diagnoses were significantly less frequent after the earthquakes; and (iv) the treatment patterns were comparable, while the crude proportions of recorded outcomes did not differ significantly between groups. However, outcome comparisons should be interpreted cautiously because of the shorter follow-up duration in the post-earthquake group.

Studies investigating the relationship between major natural disasters and AAV are limited and have yielded inconsistent findings. Following the Great Hanshin-Awaji/Kobe Earthquake, a higher regional burden of MPO-ANCA-associated vasculitis with respiratory and renal involvement was reported [9]. Similarly, the Great East Japan Earthquake and tsunami were associated with an increased incidence of microscopic polyangiitis, higher BVASs, and poorer survival outcomes [10]. In contrast, a study conducted after the Christchurch Earthquake found no significant change in AAV incidence [11]. In the present study, the crude number of newly diagnosed AAV cases was numerically higher after the earthquakes; however, this difference did not reach statistical significance. Because this was not a population-based study, the underlying population at risk and the corresponding denominator could not be reliably determined. Moreover, deaths, population displacement, migration, disrupted healthcare access, and changes in referral pathways may have substantially altered the number and characteristics of patients presenting to the participating centers after the earthquakes. Therefore, the crude numbers of newly diagnosed AAV cases observed in this study cannot be considered estimates of disease incidence or used to infer a true increase or decrease in disease occurrence. Rather, these findings should be interpreted solely as a descriptive overview of the diagnostic activity at major tertiary referral centers serving the earthquake-affected provinces.

Environmental changes following natural disasters have been suggested to play a role in the development or clinical manifestation of autoimmune diseases [16]. In particular, factors such as intense psychological stress, an increased frequency of infections, and exposure to environmental particulate matter after earthquakes have been identified as potential triggers capable of influencing the immune system [10,17]. Following the 6 February 2023 earthquakes, extensive structural destruction resulted in prolonged debris removal activities across the affected region, exposing a large population to varying levels of dust and particulate matter [18]. Silica exposure has been epidemiologically associated with the development of AAV, while experimental evidence suggests that silica particles may promote innate immune activation through NLRP3 inflammasome activation and subsequent interleukin-1β release [19,20]. In genetically susceptible individuals, this inflammatory environment may promote neutrophil activation, dysregulated immune responses against neutrophil antigens, and subsequent ANCA-mediated vascular inflammation [5,6]. Infections and severe psychological stress may further contribute through systemic inflammation, neutrophil activation, and altered immune regulation [5,17]. Thus, the combined effects of particulate exposure, infections, and psychological stress, following a major disaster, may provide a biologically plausible environment for the development or clinical manifestation of AAV in susceptible individuals. Observations reported after the Kobe Earthquake and the Great East Japan Earthquake have also provided findings supporting a possible association between post-disaster environmental exposures and AAV [9,10]. However, the present findings do not allow a causal inference regarding the role of post-earthquake environmental and psychosocial factors in the development of AAV. Given the absence of population-based incidence data and individual-level exposure information, the potential contribution of these factors requires further evaluation in larger long-term studies with reliable denominator and exposure data.

Although studies evaluating the development of AAV following earthquakes are limited, some have also examined the disease severity and patterns of clinical involvement. Following the Kobe Earthquake, patients with MPO-ANCA-associated vasculitis were reported to have more severe renal and pulmonary involvement, whereas a study conducted after the Great East Japan Earthquake reported higher BVASs and poorer survival among patients diagnosed with MPA in the post-earthquake period [9,10].

The clinical phenotype of AAV appeared broadly similar before and after the earthquakes. GPA remained the predominant subtype, and PR3-ANCA positivity was the most common serological pattern in both periods. In addition, renal, pulmonary, upper respiratory tract, neurological, cutaneous, musculoskeletal, ocular, gastrointestinal, and cardiac involvement did not differ significantly between groups. The BVASs and most inflammatory, hematological, renal, and biochemical parameters were also comparable. These findings suggest that within the limits of this tertiary-care cohort, the post-earthquake period was not associated with a clearly different baseline AAV phenotype or higher disease activity at diagnosis.

A notable finding of the present study was the significant reduction in biopsy-confirmed diagnoses after the earthquakes. However, this reduction was not specific to renal biopsy, as the kidney biopsy rates were similar in the pre-earthquake and post-earthquake groups (25.9% vs. 23.2%), and the overall distribution of biopsy sites did not differ significantly between the two periods. Potential explanations for the lower overall rate of histopathological confirmation include post-disaster disruption of hospital infrastructure and procedural services, reduced access to pathology services, altered referral pathways, difficulties in patient transportation and follow-up, and prioritization of urgent healthcare needs. In patients presenting with severe or organ-threatening manifestations, treatment may also have been initiated based on clinical, serological, and imaging findings when tissue confirmation was not readily feasible or when delaying treatment was considered clinically inappropriate.

Reduced histopathological confirmation may have important clinical implications. Biopsy can increase the diagnostic certainty, assist in excluding infections, malignancies, and other conditions that may mimic AAV, and provide organ-specific pathological information [2]. In patients with renal involvement, histopathological evaluation may also help distinguish active inflammatory lesions from chronic damage and provide information relevant to prognosis and treatment planning. Therefore, higher reliance on clinical and serological findings without tissue confirmation may increase the diagnostic uncertainty and limit pathological characterization of the disease. However, because the reasons for performing or foregoing biopsy were not recorded, the present study cannot determine whether the lower biopsy-confirmation rate affected the diagnostic accuracy, therapeutic decisions, or clinical outcomes. The proposed explanations and implications should therefore be considered hypotheses requiring further investigation.

The treatment approaches were generally comparable between the pre- and post-earthquake periods. The crude proportions of the recorded outcomes, including remission, relapse, dialysis requirement, hospitalization, intensive care unit admission, and mortality, also did not differ significantly between the groups. However, these findings should be interpreted with substantial caution because the median follow-up duration was markedly longer in the pre-earthquake group than in the post-earthquake group (52 vs. 14 months). Relapse, dialysis requirement, and mortality are time-dependent outcomes that accumulate with increasing observation time, and the shorter follow-up may have resulted in under-ascertainment of these events in the post-earthquake group. Therefore, the absence of statistically significant differences in crude outcome proportions should not be considered evidence of comparable long-term prognosis between the two periods.

This study has several strengths. To the best of our knowledge, it is the first multicenter study to evaluate newly diagnosed AAV cases before and after the 6 February 2023 Kahramanmaraş earthquakes. The study included major tertiary referral centers from provinces severely affected by the disaster and provided real-world data on a rare but potentially life-threatening autoimmune disease in a post-disaster setting. In addition, the cohort included 127 newly diagnosed AAV patients, exceeding the sample sizes of previous earthquake-related AAV studies [9,10,11]. The study also assessed a broad range of clinical variables, including the AAV subtype, ANCA specificity, organ involvement, disease activity, laboratory findings, treatment patterns, biopsy confirmation, and recorded outcomes.

Several limitations should be acknowledged. First, the retrospective design precludes causal inference. Second, this was not a population-based incidence study; therefore, the true incidence rates could not be calculated. Post-earthquake fatalities, migration, displacement, changes in healthcare access, and referral bias may have affected the number of patients diagnosed at participating centers. Third, individual-level earthquake-related exposures, including debris dust, silica exposure, infections, housing conditions, displacement, and psychological stress, were not available. Therefore, potential biological mechanisms linking disaster-related exposures and AAV onset could not be directly evaluated. In addition, detailed renal parameters, including proteinuria and microscopic hematuria, were not included in the predefined multicenter dataset and could not be retrospectively obtained in a complete and standardized manner across all participating centers. Finally, the substantially shorter follow-up duration in the post-earthquake group limits the comparison of time-dependent outcomes and precludes conclusions regarding comparable long-term prognosis.

## 5. Conclusions

In conclusion, this multicenter tertiary-care cohort showed no statistically significant difference in the crude numbers of newly diagnosed AAV cases or in the baseline clinical phenotype between the pre- and post-earthquake periods. However, because the population denominator, healthcare accessibility, referral pathways, and migration patterns changed substantially after the earthquakes, the observed case numbers should not be interpreted as incidence estimates or as evidence of a true change in disease occurrence. The AAV subtype distribution, ANCA serology, organ involvement, disease activity, and treatment patterns were largely comparable. However, biopsy-confirmed diagnoses decreased significantly after the earthquakes, suggesting possible changes in diagnostic healthcare pathways during the post-disaster period. Accordingly, these findings should be regarded as a descriptive regional overview of newly diagnosed AAV cases recorded at tertiary referral centers rather than as a population-level assessment of disease incidence. Larger population-based studies with reliable denominator data are needed to determine whether the occurrence of AAV truly changed following the disaster.

## Figures and Tables

**Table 1:** Comparison of demographic and clinical characteristics between the pre- and post-earthquake periods (*n* = 127).

Variable	Category	Pre-Earthquake (*n* = 58)	Post-Earthquake (*n* = 69)	*p* Value	Effect Size	Test
Continuous Variables						
Age (years)		50.24 ± 15.58	52.72 ± 16.73	0.392	—	*t*-test
Age at diagnosis (years)		45.93 ± 15.51	51.19 ± 16.85	0.072	—	*t*-test
Body mass index (kg/m^2^)		27.15 ± 5.24	26.46 ± 4.45	0.571	—	*t*-test
Follow-up duration (months)		52 (2–78)	14 (1–60)	<0.001	r = 0.76	MWU
Categorical Variables						
Sex	Female	28 (48.28%)	39 (56.52%)	0.354	—	Pearson’s chi-square test
	Male	30 (51.72%)	30 (43.48%)			
Smoking status	No	29 (78.38%)	39 (81.25%)	0.956	—	Yates’ continuity-corrected chi-square test
	Yes	8 (21.62%)	9 (18.75%)			
AAV subtype	GPA	44 (75.86%)	49 (71.01%)	0.412	—	Pearson’s chi-square test
	MPA	5 (8.62%)	11 (15.94%)			
	EGPA	6 (10.34%)	8 (11.59%)			
	Unclassifiable	3 (5.17%)	1 (1.45%)			
ANCA serology	PR3-ANCA	33 (56.90%)	38 (55.07%)	0.351	—	Pearson’s chi-square test
	MPO-ANCA	12 (20.69%)	20 (28.99%)			
	Double-positive	4 (6.90%)	1 (1.45%)			
	ANCA-negative	9 (15.52%)	10 (14.49%)			
Biopsy-confirmed diagnosis	No	17 (29.31%)	37 (53.62%)	0.010	V = 0.25	Yates’ continuity-corrected chi-square test
	Yes	41 (70.69%)	32 (46.38%)			
Biopsy site	No biopsy	17 (29.31%)	37 (53.62%)	0.080	—	Pearson’s chi-square test
	Kidney	15 (25.86%)	16 (23.19%)			
	Lung	12 (20.69%)	9 (13.04%)			
	Skin	5 (8.62%)	3 (4.35%)			
	Upper respiratory tract	8 (13.79%)	4 (5.80%)			
	Other	1 (1.72%)	0 (0.00%)			
Participating center	Malatya	21 (36.21%)	29 (42.03%)	0.925	—	Pearson’s chi-square test
	Kahramanmaraş	13 (22.41%)	14 (20.29%)			
	Hatay	18 (31.03%)	20 (28.99%)			
	Adıyaman	6 (10.34%)	6 (8.70%)			

Data are presented as the mean ± standard deviation (SD) for normally distributed variables, the median (minimum–maximum) for non-normally distributed variables, and *n* (%) for categorical variables. Effect sizes were calculated as r = |Z|/√N for the Mann–Whitney U test and Cramér’s V for categorical variables. The effect size interpretation was as follows: negligible (<0.20), small (0.20–0.49), moderate (0.50–0.79), and large (≥0.80). AAV, ANCA-associated vasculitis; GPA, granulomatosis with polyangiitis; MPA, microscopic polyangiitis; EGPA, eosinophilic granulomatosis with polyangiitis; ANCA, antineutrophil cytoplasmic antibody; PR3, proteinase 3; MPO, myeloperoxidase; MWU, Mann–Whitney U test.

**Table 2:** Comparison of organ involvement patterns between the pre- and post-earthquake periods.

Variable	Category	Pre-Earthquake *n* (%)	Post-Earthquake *n* (%)	*p* Value	Test
Renal involvement	No	27 (46.55)	30 (43.48)	0.729	Pearson’s chi-square test
	Yes	31 (53.45)	39 (56.52)		
Pulmonary involvement	No	10 (17.24)	14 (20.29)	0.834	Yates’ continuity-corrected chi-square test
	Yes	48 (82.76)	55 (79.71)		
Upper respiratory tract involvement	No	19 (32.76)	35 (50.72)	0.063	Yates’ continuity-corrected chi-square test
	Yes	39 (67.24)	34 (49.28)		
Neurological involvement	No	46 (79.31)	55 (79.71)	1.000	Yates’ continuity-corrected chi-square test
	Yes	12 (20.69)	14 (20.29)		
Cutaneous involvement	No	44 (75.86)	55 (79.71)	0.759	Yates’ continuity-corrected chi-square test
	Yes	14 (24.14)	14 (20.29)		
Musculoskeletal involvement	No	37 (63.79)	48 (69.57)	0.618	Yates’ continuity-corrected chi-square test
	Yes	21 (36.21)	21 (30.43)		
Ocular involvement	No	52 (89.66)	60 (86.96)	0.847	Yates’ continuity-corrected chi-square test
	Yes	6 (10.34)	9 (13.04)		
Gastrointestinal involvement	No	57 (98.28)	66 (95.65)	0.625	Fisher’s exact test
	Yes	1 (1.72)	3 (4.35)		
Cardiac involvement	No	56 (96.55)	66 (95.65)	1.000	Fisher’s exact test
	Yes	2 (3.45)	3 (4.35)		
Other manifestations	No	57 (98.28)	67 (97.10)	1.000	Fisher’s exact test
	Yes	1 (1.72)	2 (2.90)		

Data are presented as *n* (%). Yates’ continuity-corrected chi-square test and Fisher’s exact test were used where appropriate. Effect sizes were not calculated because no statistically significant differences were observed.

**Table 3:** Comparison of laboratory parameters and BVASs between the pre- and post-earthquake periods (*n* = 127).

Variable	Pre-Earthquake *n* (%)	Post-Earthquake *n* (%)	*p* Value	Effect Size	Test
BVAS	15.10 ± 8.55	15.43 ± 10.15	0.844	d = 0.04	*t*-test
ESR (mm/h)	57.45 ± 31.98	56.41 ± 35.83	0.864	d = 0.03	*t*-test
White blood cell count (×10^3^/µL)	11,428.79 ± 4728.12	10,110.55 ± 3963.67	0.090	d = 0.31	*t*-test
Hemoglobin (g/dL)	11.52 ± 2.32	11.53 ± 2.81	0.980	d = 0.01	*t*-test
Albumin (g/dL)	3.51 ± 0.56	3.49 ± 0.57	0.874	d = 0.03	*t*-test
CRP (mg/L)	61.50 (2–231)	48.00 (3–288)	0.647	r = 0.04	MWU
Serum creatinine (mg/dL)	0.95 (0.50–7.80)	1.10 (0.48–12.10)	0.794	r = 0.02	MWU
ALT (U/L)	18 (7–303)	20 (5–143)	0.746	r = 0.03	MWU
AST (U/L)	18.50 (7–348)	19 (8–86)	0.905	r = 0.01	MWU
Platelet count (×10^3^/µL)	347.50 (147–658)	287.00 (163–695)	0.029	r = 0.19	MWU

Data are presented as the mean ± standard deviation (SD) for normally distributed variables and the median (minimum–maximum) for non-normally distributed variables. Effect sizes were calculated using Cohen’s d for the independent-samples *t* test and r = |Z|/√N for the Mann–Whitney U test. BVAS, Birmingham Vasculitis Activity Score; ESR, erythrocyte sedimentation rate; CRP, C-reactive protein; ALT, alanine aminotransferase; AST, aspartate aminotransferase; *t* test, independent-samples *t* test; MWU, Mann–Whitney U test.

**Table 4:** Comparison of treatment approaches and clinical outcomes between the pre- and post-earthquake periods.

Variable	Category	Pre-Earthquake *n* (%)	Post-Earthquake *n* (%)	*p* Value	Test
Pulse glucocorticoid therapy	No	17 (29.31)	21 (30.43)	1.000	Yates’ continuity-corrected chi-square test
	Yes	41 (70.69)	48 (69.57)		
Cyclophosphamide	No	21 (36.21)	34 (49.28)	0.193	Yates’ continuity-corrected chi-square test
	Yes	37 (63.79)	35 (50.72)		
Rituximab	No	25 (43.10)	25 (36.23)	0.430	Pearson’s chi-square test
	Yes	33 (56.90)	44 (63.77)		
Azathioprine	No	29 (50.00)	46 (66.67)	0.085	Yates’ continuity-corrected chi-square test
	Yes	29 (50.00)	23 (33.33)		
Methotrexate	No	50 (86.21)	65 (94.20)	0.219	Yates’ continuity-corrected chi-square test
	Yes	8 (13.79)	4 (5.80)		
Mycophenolate mofetil	No	37 (63.79)	46 (66.67)	0.879	Yates’ continuity-corrected chi-square test
	Yes	21 (36.21)	23 (33.33)		
Plasmapheresis	No	51 (87.93)	63 (91.30)	0.741	Yates’ continuity-corrected chi-square test
	Yes	7 (12.07)	6 (8.70)		
Hospitalization	No	7 (12.07)	11 (15.94)	0.713	Yates’ continuity-corrected chi-square test
	Yes	51 (87.93)	58 (84.06)		
Intensive care unit admission	No	49 (84.48)	62 (89.86)	0.522	Yates’ continuity-corrected chi-square test
	Yes	9 (15.52)	7 (10.14)		
Dialysis requirement	No	50 (86.21)	62 (89.86)	0.720	Yates’ continuity-corrected chi-square test
	Yes	8 (13.79)	7 (10.14)		
Remission	No	8 (13.79)	11 (15.94)	0.929	Yates’ continuity-corrected chi-square test
	Yes	50 (86.21)	58 (84.06)		
Relapse	No	38 (65.52)	50 (72.46)	0.514	Yates’ continuity-corrected chi-square test
	Yes	20 (34.48)	19 (27.54)		
Mortality	No	56 (96.55)	69 (100.00)	0.207	Fisher’s exact test
	Yes	2 (3.45)	0 (0.00)		

Data are presented as *n* (%). Effect sizes were not calculated because no statistically significant differences were observed.

**Table 5:** Correlations between BVASs and laboratory parameters in the overall cohort and subgroups.

Variables	Overall Cohort (*n* = 127)		Pre-Earthquake (*n* = 58)		Post-Earthquake (*n* = 69)	
	r	*p* Value	r	*p* Value	r	*p* Value
Inflammatory Markers						
CRP (mg/L)	+0.423	<0.001	+0.418	0.001	+0.428	<0.001
ESR (mm/h)	+0.295	0.001	+0.262	0.047	+0.313	0.009
Renal function						
Serum creatinine (mg/dL)	+0.426	<0.001	+0.428	0.001	+0.427	<0.001
Hematological Parameters						
Hemoglobin (g/dL)	−0.371	<0.001	−0.265	0.044	−0.440	<0.001
White blood cell count (×10^3^/µL)	+0.358	<0.001	+0.432	0.001	+0.312	0.009
Platelet count (×10^3^/µL)	+0.198	0.026	+0.238	0.072	+0.172	0.159
Nutritional Status						
Albumin (g/dL)	−0.374	<0.001	−0.308	0.019	−0.413	<0.001
Liver enzymes						
ALT (U/L)	−0.041	0.643	+0.070	0.601	−0.125	0.308
AST (U/L)	−0.019	0.830	+0.174	0.191	−0.140	0.251

r, Spearman’s rank correlation coefficient. Correlation strength was interpreted as follows: negligible (<0.20), weak (0.20–0.39), moderate (0.40–0.69), and strong (0.70–0.89). BVAS, Birmingham Vasculitis Activity Score; CRP, C-reactive protein; ESR, erythrocyte sedimentation rate; ALT, alanine aminotransferase; AST, aspartate aminotransferase.

## Data Availability

The data presented in this study are not publicly available due to privacy and ethical restrictions related to patient-level clinical data. Anonymized data may be made available from the corresponding author upon reasonable request and with appropriate institutional approval.

## Abbreviations

The following abbreviations are used in this manuscript:

AAV	ANCA-associated vasculitis
ACR/EULAR	American College of Rheumatology/European Alliance of Associations for Rheumatology
ALT	Alanine aminotransferase
ANCA	Antineutrophil cytoplasmic antibody
AST	Aspartate aminotransferase
BMI	Body mass index
BVAS	Birmingham Vasculitis Activity Score
CRP	C-reactive protein
EGPA	Eosinophilic granulomatosis with polyangiitis
ENT	Ear, nose, and throat
ESR	Erythrocyte sedimentation rate
GPA	Granulomatosis with polyangiitis
MPA	Microscopic polyangiitis
MPO	Myeloperoxidase
PR3	Proteinase 3
SD	Standard deviation
WBC	White blood cell
